# Trends in Activity Limitations From an International Perspective: Differential Changes Between Age Groups Across 30 Countries

**DOI:** 10.1177/08982643221141123

**Published:** 2022-11-25

**Authors:** Johannes Beller, Marc Luy, Guido Giarelli, Enrique Regidor, Lourdes Lostao, Juliane Tetzlaff, Siegfried Geyer

**Affiliations:** 1 Medical Sociology Unit, 9177 Hannover Medical School, Germany; 2 Vienna Institute of Demography, 111578 Austrian Academy of Sciences, Austria; 3 Department of Health Sciences, 16734 University “MAGNA GRAECIA” Catanzaro, Italy; 4 Department of Public Health & Maternal and Child Health, 16756 Complutense University of Madrid, Spain; 5 Department of Sociology, Public University of Navarre, Germany

**Keywords:** disability, functional limitations, morbidity, trends

## Abstract

**Objectives:** Examine trends in limitations among young (15–39), middle-aged (40–64) and older age-groups (>=65) and their socioeconomic differences. **Methods:** Population-based European Social Survey data (*N* = 396,853) were used, covering 30 mostly European countries and spanning the time-period 2002–2018. Limitations were measured using a global activity limitations indicator. **Results:** Age-differential trends in limitations were found. Activity limitations generally decreased in older adults, whereas trends varied among younger and middle-aged participants, with decreasing limitations in some countries but increasing limitations in others. These age-differential trends were replicated across limitation severity and socioeconomic groups; however, stronger limitation increases occurred regarding less-severe limitations. **Discussion:** Functional health has improved in older adults. Contrarily, the increasing limitations in younger and middle-aged individuals seem concerning, which were mostly observed in Western and Northern European countries. Given its public health importance, future studies should investigate the reasons for this declining functional health in the young and middle-aged.

## Introduction

Activity limitations are difficulties an individual might have in executing everyday activities. In older adults, activity limitations are mostly related to Activities of Daily Living (ADL) and Instrumental Activities of Daily Living (IADL) limitations like difficulties in dressing, walking or shopping. In middle-aged and younger adults, however, activity limitations are most strongly related to work activity limitations, such as prolonged sick leave or inability to work (Cabrero-García et al., 2020, 2021; Jagger et al., 2010). Activity limitations pose a significant burden to health and well-being (Üstün et al., 2003): In large, longitudinal studies limitations have been shown to predict decreased well-being, increased mental health problems, onset of disease, cognitive decline and mortality (e.g. Farragher et al., 2006; Heine et al., 2019; Ruano et al., 2017). Activity limitations are also associated with the inability to work, and the need to use supportive technology or long-term care (Mitra et al., 2017). As limitation rates increase with age, prevalences of limitations are highest in older adults. However, a large part of younger and middle-aged adults are also affected (Beller & Epping, 2020; Karvonen-Gutierrez & Strotmeyer, 2020; Verbrugge, 2016; von Bonsdorff & Rantanen, 2011). In the last years, several trends which might lead to increasing limitations have been identified, including the rise of obesity in all age groups, the decreasing mental health among youth and younger adults, advancing medical therapies in older adults and an increase of sedentary behaviour at the workplace (Malik et al., 2013; Owen et al., 2020; Schoeni et al., 2008; Weinberger et al., 2018). These large-scale trends might have also led to recent temporal changes in activity limitations. Given the importance of activity limitations to individual and societal functioning, there is thus a need to study time trends in limitations.

### Previous Literature

Numerous seminal studies have already analysed trends in activity limitations, with most studies focussing on trends in older adults, aged 65 years and older (Crimmins, 2004; Robine & Jagger, 2017). As an early example, Verbrugge summarized health trends in the United States up until the 1980s, finding evidence for increasing disease prevalence and disability for American adults aged 45 years and older (Verbrugge, 1989). About two decades later, Crimmins reviewed health trends in US older adults, aged 65 years and older until the 1990s. It was found that most measures of health, including disability and severe disability, have improved over time in older adults (Crimmins, 2004). Recently, however, Jagger and colleagues analysed trends in disability-free life expectancy at age 65 in England and found substantial increases in mild limitations from 1991 to 2011 (Jagger et al., 2016). Analysing trends cross-nationally, Lafortune and Balestat reviewed trends in severe disability among older adults, aged 65 years and older, in 12 OECD countries from the 1980s up until 2005. The authors found that severe disability has declined in some countries like Finland but remained the same or even increased in other countries like Canada (Lafortune & Balestat, 2007). As a last example, Lee et al. (2020) studied trends in disability among adults aged 60 years and older in 20 countries from 2004 to 2014. Results in most countries pointed to either significantly decreasing or non-significant trends in older adults. However, similar, to Lafortune and Balestat, the authors found widely diverging trends in disability across countries, noting that future research on this topic is needed. Therefore, while most previous studies have found evidence for decreasing trends in limitations among older adults, other studies have emphasized cross-national differences in these trends.

Despite the fact that a large part of young and middle-aged adults are also affected by activity limitations, only some studies have recently empirically distinguished limitation trends in younger, middle-aged and older age groups. For example, Freedman and colleagues have analysed trends in limitations among middle-aged (ages 55–64) and older adults (65+) in the United States (Freedman et al., 2013). Using multiple national surveys, they found that limitation rates were generally stable or declined among older adults but modestly increased among the middle-aged. Among the studies that compared trends in different age groups internationally, Beller and colleagues focused on generational differences in disability and found that disability rates were increasing on the birth cohort level in multiple European countries, such that younger cohorts born after about 1960 had increasing levels of limitations (Beller & Epping, 2020; Beller et al., 2019). To our knowledge, only one additional study, Verropoulou and Tsimbos (2017), analysed trends in limitations in middle-aged (ages 50–64) and older adults (65+) in multiple countries. The authors found that limitation rates decreased over time in older adults of most studied European countries. Results were more mixed in middle-aged adults, where the authors observed decreasing trends in some countries but increasing trends in others. Thus, although some studies have addressed an important issue by comparing health trends in limitations across several countries, previous studies have focused mostly on the older populations. Clearly, more evidence regarding the cross-national trends in limitations among different age groups, including younger adults, is needed. It seems especially important to include even more countries, because the onset of limitations strongly depends on contextual factors, and thus, trends might differ between countries (Üstün et al., 2003). Analysing trends in functional limitations seems also important from a theoretical perspective. Several theories have been proposed to describe how population level morbidity will develop over time, including the compression of morbidity hypothesis and the expansion of morbidity hypothesis (Fries, 1980; Fries et al., 2011; Gruenberg, 1977; Kramer, 1980). Increasing rates of disability in middle aged or older adults, as observed in some studies, might suggest a possible expansion of morbidity in the future.

Additionally, there are differences in the prevalence of limitations and trends in limitations between socioeconomic groups (Mackenbach, 2019). Similar to the literature on limitation trends, most studies on inequalities in limitations have been conducted among older adults living in the US. Generally, limitation prevalences are found to be higher among the less-educated and the less-affluent, and most studies point to a widening of inequalities between socioeconomic groups over time (e.g. Melzer et al., 2001; Schoeni et al., 2005; Tsai, 2016). Fewer studies on socioeconomic differences in limitations have been conducted among European samples and younger age groups (see, however, for example, Martin et al., 2012; Moe & Hagen, 2011; von dem Knesebeck et al., 2017). Of note, one recent study comprehensively investigated social inequalities in limitations in 26 European countries from 2002 to 2017 among people aged 30–79 years (Rubio-Valverde et al., 2021). The authors found that socioeconomic differences between groups, as measured by educational attainment, tended to remain the same or increase depending on the data source and the country considered (Rubio-Valverde et al., 2021).

### Aims of the Current Study

The current study aims to further investigate time trends in functional limitations. It goes beyond most previous studies by differentiating trends in activity limitations between different age-groups, including young, middle-aged and older age-groups, by investigating how these trends in age-groups differ according to education and income difficulties, and by using a large (*N* = 396,853; n_Young_ = 139,000; n_Middle_ = 167,902; n_Older_ = 89,951), cross-national (*N* = 30 countries), population-based sample spanning the time period from 2002 to 2018. This sample not only includes participants from Western and Northern Europe, which have been frequently studied in previous research, but also includes countries from Eastern and Southern Europe, as well as Israel and Russia. Only some studies have empirically compared trends in limitations across different age groups and it remains unclear how generalizable the finding of contrasting trends in different age groups really is. Furthermore, studies are missing that examine socio-economic differences in these age-dependent trends. Thus, the current study advances on similar previous studies like the one from Beller and Epping (2020) by investigating a larger set of countries (30 vs. 15 countries), by explicitly investigating trends in different age groups instead of birth cohorts and by also investigating socio-economic differences in these age-dependent trends. Thereby, the current study clarifies (a) how activity limitations have changed over time in young, middle-aged and older individuals, (b) whether the same trends can be found for overall and severe limitations and for different socio-economic subgroups and (c) whether these trends differ or are similar across a diverse set of countries.

## Methods

### Sample

Data were drawn from the public release of the cumulative European Social Survey (ESS) that aims to provide comparative data on attitudes, beliefs and behaviour patterns of the various populations in Europe. We used data from the 30 countries (Austria, Belgium, Bulgaria, Switzerland, Cyprus, Czechia, Germany, Denmark, Estonia, Spain, Finland, France, Great Britain, Greece, Croatia, Hungary, Ireland, Israel, Iceland, Italy, Lithuania, Netherlands, Norway, Poland, Portugal, Russia, Sweden, Slovenia, Slovakia, Ukraine) that participated in at least three of the nine waves of the survey (sampled in 2002, 2004, 2006, 2008, 2010, 2012, 2014, 2016 and 2018). Of the 30 countries, 15 countries (Belgium, Switzerland, Germany, Spain, Finland, France, Great Britain, Hungary, Ireland, Netherlands, Norway, Poland, Portugal, Sweden and Slovenia) participated in every wave, whereas the other 15 countries participated in between three to eight waves. The ESS provides population-based samples of non-institutionalized participants aged 15 years and older with the interviews conducted face-to-face at the respondent’s place of residence (Jowell et al., 2007). For each country, sampling designs are developed by an expert panel in cooperation with national survey teams. All selection processes are based on random probability samples. Response rates vary strongly between countries, for example, in 2002, between 34% in Switzerland and 80% in Greece, and in 2018, between 28% in Germany and 69% in Bulgaria (further information on response rates in the ESS is reported by Koen Beullens, 2018). Various sampling designs are used to ensure an as representative cross-national sampling process as possible, including stratified random sampling and multi-stage random sampling. Importantly, however, people in institutional care are not included in the sample. All countries use the same target population, individuals of at least 15 years of age living permanently in a private household. Accordingly, sample designs in the ESS may vary across countries, but are implemented to make estimates between countries as comparable as possible. All samples are independent from each other from wave to wave. Interviews may only be conducted with the sampled individuals. Substitution or proxy interviews are not allowed. To account for sampling differences between countries, all reported results (except the descriptive tables reported only in the appendix) are weighted according to the design weights provided by the ESS. Participants provided informed consent and all procedures were in accordance with the ethical standards of the institutional research committee and with the 1964 Helsinki declaration and its later amendments. After omitting participants with missing values listwise (*n* = 15,775; less than 4% of the whole sample), a final sample with *N* = 396,853 participants resulted (Austria: *n* = 12,745; Belgium: *n* = 15,822; Bulgaria: *n* = 10,341; Switzerland: *n* = 15,147; Cyprus: *n* = 4983; Czechia: *n* = 16,243; Germany: *n* = 25,162; Denmark: *n* = 12,158; Estonia: *n* = 15,162; Spain: *n* = 16,371; Finland: *n* = 17,778; France: *n* = 13,580; Great Britain: *n* = 19,397; Greece: *n* = 9619; Croatia: *n* = 4692; Hungary: *n* = 14,473; Ireland: *n* = 19,567; Israel: *n* = 13,969; Iceland: *n* = 2959; Italy: *n* = 7052; Lithuania: *n* = 9507; Netherlands: *n* = 16,570; Norway: *n* = 14,552; Poland: *n* = 15,225; Portugal: *n* = 15,587; Russia: *n* = 11,744; Sweden: *n* = 15,732; Slovenia: *n* = 11,968; Slovakia: *n* = 9397; and Ukraine: *n* = 9351).

### Measures

Limitations were measured with a single item, similar to the Global Activity Limitation Indicator (GALI) (van Oyen et al., 2006), using the question: ‘Are you hampered in your daily activities in any way by any longstanding illness, disability, infirmity or mental health problem’? The participant could choose to respond with one of three different answers: ‘yes, a lot’, ‘yes, to some extent’ or ‘no’. For our analysis, we dichotomized the response 2 ways: First, in ‘yes, a lot’ or ‘yes, to some extent’ versus ‘no’ to measure overall limitations; and, second in ‘yes, a lot’ versus ‘yes, to some extent’ and ‘no’ to measure severe limitations. Several previous studies have found that single item indicators of limitations provide cost-effect alternatives to longer disability measures (e.g. Berger et al., 2015; Van Oyen et al., 2018). Additionally, this limitations measure has been successfully applied in several previous studies (e.g. Beller & Epping, 2020; Campos-Matos et al., 2016; Nicholson et al., 2010; Álvarez-Gálvez & Jaime-Castillo, 2018).

The time period (year of survey wave) was included as a metric predictor to estimate the average change per year. Although this strategy might obfuscate fluctuations between survey years, this approach enables the better comparison of general trends both across countries and age groups, which was the main goal of the study. Years of education were measured by asking participants: ‘About how many years of education have you completed, whether full-time or part-time’? Responses were dichotomized into ‘Fewer Years of Education’ for responses equal to or lower than 12 years of education (the median in the sample); conversely, responses higher than 12 years of education were coded as ‘More Years of Education’. As an alternative operationalization of education, educational attainment was measured according to the International Standard Classification of Education, as operationalized in the ESS. Individual levels of educational attainment were dichotomized into ‘Lower Education’ for respondents with up to the secondary level; conversely, respondents with post-secondary levels of education were classified as having a ‘Higher Education’. Income difficulties are measured by asking respondents: ‘Which of the descriptions on this card comes closest to how you feel about your household’s income nowadays’? Participants could choose to respond with ‘Living comfortably on present income’, ‘Coping on present income’, ‘Finding it difficult on present income’ or ‘Finding it very difficult on present income’, with the latter two answer categories being combined to denote ‘Income Difficulties’ and the first two answer categories denoting ‘No Income Difficulties’. Furthermore, age and gender (male or female) were included.

### Data Analysis

First, descriptive statistics of all variables according to age groups (young age = ages 15–39; middle age = ages 40–64; and old age = ages 65 and above) are reported. Then, to determine general trends across countries, multilevel logistic regression analyses are conducted within the respective age groups, with a random intercept by country predicting limitations via age (scaled in 10 years, such that an increase by 1 on the metric corresponds to an increase in 10 years of age), gender (0 = male; 1 = female) and time period (scaled in 10 years, such that an increase by 1 on the metric corresponds to an increase in 10 years of time). General trends were also analysed in stratified samples according to education and income difficulties. A similar data analytic strategy was used to study trends within countries. However, in this case, simple logistic regression analysis was used predicting limitations via age (in 10 years), gender (0 = male; 1 = female) and time period (in 10 years). Thus, the time period coefficient can be interpreted as the average change in the odds of having limitations over a time period of 10 years across countries (multilevel logistic regression analyses) or within countries (simple logistic regression analyses depicted via plots). All results are weighted according to the design weights provided by the ESS.

## Results

**Figure A1.**
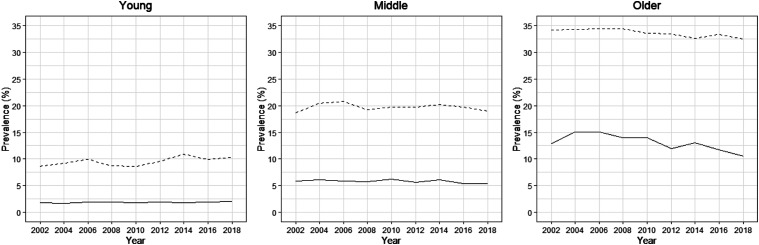
Raw prevalence in overall limitations (Dashed Line) and severe limitations (Solid Line) over time in young (left; ages 15–39), middle-aged (Center; ages 40–64) and older adults (Right; ages 65+) (European social survey data from 2002–2018) *Note.* Multiple countries, such as Hungary, did not participate in every survey wave. As such, the displayed raw overall prevalence estimates for each year depend on the countries participating in that wave and interpretation about trends should mostly be based on the global trends analysed via multilevel analysis presented in Table 2 and the trends at the national level presented in Figure 1 and Figure 2.

Overall, after weighting, participants were on average 47.31 (*SD* = 18.34) years old, with 53% being female. On average, 25% reported having any limitation, of which 6% was reported as a severe limitation. As depicted in Table 1, overall limitation and severe limitation prevalence was higher in older age-groups. However, limitation prevalence varied strongly across countries, as seen in Table A1, with participants from Ukraine having among the highest prevalence of limitations (41% with overall limitations of which 10% were severe limitations) and participants from Italy having among the lowest prevalence (14% with overall limitations of which 2% were severe limitations). More detailed descriptive statistics regarding time periods, countries, age-groups and overall and severe limitations can be found in the Appendix Table A2, Table A3, Table A4, Table A5, Table A6, Table A7, Table A8 and Table A9. As seen in Figure A1, limitations tended to increase on a descriptive basis among the young age-group over time periods, limitations seemed to remain relatively stable in the middle-aged group and limitations tended to decrease over time in the older group.

**Table 1.** Limitations and Socio-Demographics Across Age Groups (European Social Survey Data From 2002–2018).

	Young (ages 15–39) *N* = 139000		Middle (ages 40–64) *N* = 167902		Older (ages 65+) *N* = 89951	
	%/M	SD	%/M	SD	%/M	SD
Overall limitations	11.37%	—	25.49%	—	46.53%	—
Severe limitations	1.89%	—	5.80%	—	12.99%	—
No limitations	88.63%	—	74.51%	—	53.47%	—
Age	27.62	7.14	51.64	7.09	73.47	6.50
Gender (Female)	51.78%	—	53.81%	—	54.65%	—

Next, multilevel logistic regression analyses were used to study trends over countries within age-groups adjusted for age and gender. As depicted in Table 2, among the young age group limitations significantly increased over time with ORs = 1.30 (overall limitations) and 1.17 (severe limitations), respectively. Among the middle-aged and older age groups decreasing trends among overall and severe limitations were found, with ORs of 0.90 and 0.84 among middle-aged adults as well as 0.72 and 0.62 among older adults. The general direction of trends in age-groups were similar between participants with fewer years of education and more years of education, as depicted in Table 3 (please see Table A10 for the respective results according to the alternative operationalization of education as educational attainment). However, differences emerged regarding the size of the trend effect. Increases in limitations in young adults were stronger among those with more years of education, whereas decreases in limitations in older adults were stronger among those with fewer years of education. Correspondingly, limitations increased with the largest relative effect size in younger adults with more years of education (OR = 1.51) and limitations decreased with the largest relative effect in older adults with fewer years of education (OR = 0.75). Conversely, general relative trends in age-groups were very similar between participants with income difficulties and without income difficulties, as depicted in Table 4. While significant decreases in limitations were observed among middle-aged adults with income difficulties (OR = 0.88) and no significant decreases could be observed in middle-aged adults without income difficulties (OR = 0.97), it must be noted that the confidence intervals of both coefficients overlapped, as depicted in Table 4.

**Table 2.** Multilevel Logistic Regression Results Predicting Overall and Severe Limitations Across Time (European Social Survey data From 2002–2018).

Overall limitations					Severe limitations				
Young age (ages 15–39)									
*N* = 139000	OR	CI	Z	*p*	*N* = 13900	OR	CI	Z	*p*
Age	1.29	[1.26, 1.32]	21.56	< .001	Age	1.04	[1.03, 1.04]	13.58	< .001
Gender (Female)	1.17	[1.14, 1.21]	9.53	< .001	Gender (Female)	1.11	[1.03, 1.20]	2.75	.006
Trend	1.30	[1.21, 1.39]	7.57	< .001	Trend	1.17	[1.00, 1.37]	1.97	.049

Middle age (ages 40–64)									
*N* = 167902	OR	CI	Z	*p*	*N* = 167902	OR	CI	Z	*p*
Age	1.65	[1.62, 1.68]	61.66	< .001	Age	1.05	[1.05, 1.05]	31.83	< .001
Gender (Female)	1.17	[1.14, 1.20]	13.67	< .001	Gender (Female)	1.03	[.99, 1.08]	1.55	.120
Trend	0.90	[0.86, 0.94]	−4.37	< .001	Trend	0.84	[0.77, 0.91]	−4.12	< .001

Older ages (ages 65+)									
*N* = 89951	OR	CI	Z	*p*	*N* = 89951	OR	CI	Z	*p*
Age	1.82	[1.78, 1.86]	51.18	< .001	Age	1.07	[1.06, 1.07]	40.36	< .001
Gender (Female)	1.21	[1.18, 1.25]	12.92	< .001	Gender (Female)	1.22	[1.17, 1.28]	9.16	< .001
Trend	0.72	[0.68, 0.76]	−10.72	< .001	Trend	0.62	[0.57, 0.68]	−10.49	< .001

**Table 3.** Multilevel Logistic Regression Results Predicting Overall Limitations Across Time According to Years of Education (European Social Survey Data From 2002–2018).

Fewer years of education					More years of education				
Young age (ages 15–39)									
*N* = 63317	OR	CI	Z	*p*	*N* = 75683	OR	CI	Z	*p*
Age	1.47	[1.42, 1.51]	24.43	< .001	Age	1.18	[1.14, 1.23]	8.66	< .001
Gender (Female)	1.23	[1.17, 1.29]	8.34	< .001	Gender (Female)	1.16	[1.11, 1.22]	6.52	< .001
Trend	1.24	[1.12, 1.37]	4.14	< .001	Trend	1.51	[1.38, 1.66]	8.69	< .001

Middle age (ages 40–64)									
*N* = 87439	OR	CI	Z	*p*	*N* = 80463	OR	CI	Z	*p*
Age	1.63	[1.60, 1.67]	44.82	< .001	Age	1.56	[1.52, 1.59]	35.50	< .001
Gender (Female)	1.16	[1.13, 1.20]	9.72	< .001	Gender (Female)	1.21	[1.17, 1.25]	10.83	< .001
Trend	0.96	[0.90, 1.02]	-1.21	.227	Trend	1.03	[0.96, 1.10]	0.72	.471

Older age (ages 65+)									
*N* = 65763	OR	CI	Z	*p*	*N* = 24188	OR	CI	Z	*p*
Age	1.79	[1.74, 1.84]	42.86	< .001	Age	1.73	[1.65, 1.81]	22.81	< .001
Gender (Female)	1.21	[1.17, 1.26]	11.05	< .001	Gender (Female)	1.11	[1.05, 1.18]	3.66	< .001
Trend	0.75	[0.70, 0.81]	−7.88	< .001	Trend	0.83	[0.73, 0.93]	−3.08	< .001

**Table 4.** Multilevel Logistic Regression Results Predicting Overall Limitations Across Time According to Difficulties With Income (European Social Survey Data From 2002–2018).

Income difficulties					No income difficulties				
Young age (ages 15–39)									
*N* = 33360	OR	CI	Z	*p*	*N* = 105640	OR	CI	Z	*p*
Age	1.52	[1.45, 1.59]	18.16	< .001	Age	1.19	[1.16, 1.22]	12.36	< .001
Gender (Female)	1.06	[1.00, 1.13]	1.89	.058	Gender (Female)	1.19	[1.14, 1.24]	8.62	< .001
Trend	1.32	[1.15, 1.51]	3.93	< .001	Trend	1.38	[1.27, 1.49]	7.95	< .001

Middle age (ages 40–64)									
*N* = 45328	OR	CI	Z	*p*	*N* = 122574	OR	CI	Z	*p*
Age	1.80	[1.75, 1.85]	39.73	< .001	Age	1.63	[1.60, 1.66]	48.92	< .001
Gender (Female)	1.13	[1.08, 1.17]	5.73	< .001	Gender (Female)	1.14	[1.11, 1.18]	9.61	< .001
Trend	0.88	[0.81, 0.97]	−2.70	.007	Trend	0.97	[0.91, 1.02]	−1.20	.232

Older age (ages 65+)									
*N* = 26826	OR	CI	Z	*p*	*N* = 63125	OR	CI	Z	*p*
Age	1.80	[1.72, 1.89]	24.61	< .001	Age	1.84	[1.79, 1.89]	44.90	< .001
Gender (Female)	1.23	[1.16, 1.30]	7.02	< .001	Gender (Female)	1.14	[1.10, 1.18]	7.49	< .001
Trend	0.73	[0.65, 0.83]	−4.84	< .001	Trend	0.75	[0.70, 0.81]	−8.05	< .001

**Figure 1.**
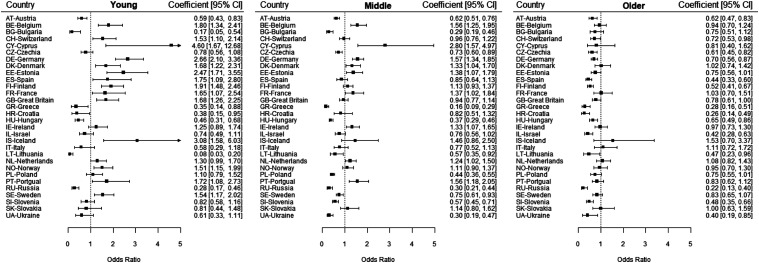
Adjusted trend coefficients regarding overall limitations within countries for young (Age 15–39; *n* = 139,000), middle (Age 40–64; *n* = 167,902) and older (Age 65+; *n* = 89,951) age-groups (European Social Survey data from 2002–2018). Note. Overall limitations in the younger age groups increased significantly in 13 countries and decreased significantly in 7 countries. Overall limitations in the middle age groups increased significantly in 9 countries and decreased significantly in 11 countries. Overall limitations in the older age groups increased significantly in 0 countries and decreased significantly in 15 countries.

**Figure 2.**
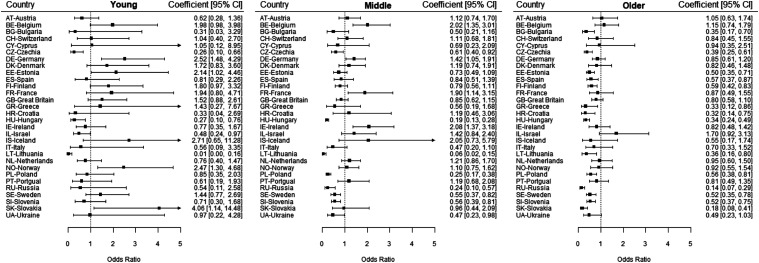
Adjusted trend coefficients regarding severe limitations within countries for young (Age 15–39; *n* = 139,000), middle (Age 40–64; *n* = 167,902) and older (Age 65+; *n* = 89,951) age-groups (European Social Survey data from 2002–2018). Note. Severe limitations in the younger age groups increased significantly in 4 countries and decreased significantly in 4 countries. Severe limitations in the middle age groups increased significantly in 4 countries and decreased significantly in 8 countries. Severe limitations in the older age groups increased significantly in 0 countries and decreased significantly in 14 countries.

Trends among age-groups varied widely within countries, as depicted in Figure 1 and Figure 2. Among the young age-group, significant increases over time in overall limitations were found in 13 countries (Belgium, Switzerland, Cyprus, Germany, Denmark, Estonia, Spain, Finland, France, Great Britain, Iceland, Norway, Portugal and Sweden), whereas significant decreases were found in 7 countries (Austria, Bulgaria, Greece, Croatia, Hungary, Lithuania and Russia). Regarding severe limitations among the young age-group, significant increases over time in severe limitations were found in 4 countries (Germany, Estonia, Norway and Slovakia), whereas significant decreases were found in 4 countries (Czechia, Hungary, Israel, and Lithuania). Among the middle-aged group, significant increases over time in overall limitations were found in 9 countries (Belgium, Cyprus, Germany, Denmark, Estonia, France, Ireland, Netherlands and Portugal), whereas significant decreases were found in 11 countries (Austria, Bulgaria, Czechia, Greece, Hungary, Lithuania, Poland, Russia, Sweden, Slovenia and Ukraine). Regarding severe limitations among the middle-aged group, significant increases over time were found in 4 countries (Belgium, Germany, France and Ireland), whereas significant decreases were found in 8 countries (Czechia, Hungary, Lithuania, Poland, Russia, Sweden, Slovenia and Ukraine). Among the older group, significant increases over time in overall limitations were found in no country, whereas significant decreases were found in 15 countries (Austria, Switzerland, Czechia, Germany, Spain, Finland, Great Britain, Greece, Croatia, Hungary, Israel, Lithuania, Russia, Slovenia and Ukraine). Regarding severe disability among the older group, significant increases over time in were found in no country, whereas significant decreases were found in 14 countries (Bulgaria, Czechia, Estonia, Spain, Finland, Greece, Croatia, Hungary, Lithuania, Poland, Russia, Sweden, Slovenia and Slovakia).

## Discussion

We examined trends in activity limitations among young (ages 15–39), middle-aged (ages 40–64) and older individuals (ages 65+) across 30 mostly European countries. We found that limitations generally decreased among older adults, including across all analysed socio-economic groups. Among middle-aged and younger adults, however, these trends were more varied: Whereas decreasing trends were found in some countries like Hungary, Czechia and Israel, increasing trends were also found in many other countries, including Germany, Belgium and France.

### Comparison With Previous Studies

These results both confirm and contradict previous studies. Most previous studies had pointed towards declining rates of limitations among older adults, aged 65 years and older (e.g. Freedman et al., 2013; Jehn & Zajacova, 2018; Verropoulou & Tsimbos, 2017). However, it had remained unclear to what degree this trend could be generalized across countries (Lee et al., 2020). In our analyses, we found significantly declining trends in most of our country-specific results, and only non-significant trends in the other cases. Additionally, these decreases could be observed in older adults with fewer and more years of education and with income difficulties and without income difficulties. Thus, our results suggest that older adults’ limitations seem to generally decline or at least not significantly increase over time in Europe, confirming most previous research. These decreases in prevalence might be seen as a first indication for morbidity compression in the older population, if the results are confirmed and extended by further studies (Fries, 1980; Fries et al., 2011). However, as life expectancy across countries has also increased over the study period, general morbidity might have extended as well. Therefore, trends in activity limitations should also be compared with trends in life expectancy to further examine the compression and expansion of morbidity hypothesis (Gruenberg, 1977), as has been already done by many previous seminal studies (e.g. Jagger et al., 2020; Spiers et al., 2011; Tawiah et al., 2021; Welsh et al., 2021).

Previous studies had also suggested that there might be differential trends in limitations between middle-aged and older age groups (Beller et al., 2019; for example, Beller, Bauersachs, et al., 2020; Freedman et al., 2013; Verropoulou & Tsimbos, 2017). Whether diverging trends could be found in multiple countries, and whether these diverging trends also apply to younger age ranges had not been systematically studied. Supporting some previous studies, it was found that increases in limitations were actually strongest among the youngest, the age group that had been studied most seldomly in previous research (e.g. Beller, Regidor, et al., 2020). However, while most previous studies had used the same age-categorization of older adults as participants aged 65 years and older, it must be noted that the operationalization of middle-aged and younger adults differs between studies, thus making exact comparisons more difficult.

Going beyond most previous studies we also differentiated between overall and severe limitations (e.g. Lee et al., 2020; Verropoulou & Tsimbos, 2017). In our results, it emerged that increases among the young were substantially stronger for overall limitations, whereas the decreases among older adults were much stronger in the case of severe limitations. Thus, less-severe activity limitations, such as difficulties with reading due to vision problems or work activity limitations seem to have increased most strongly among younger and middle-aged adults, whereas severe activity limitations, such as not being able to walk short distances or to climb stairs, might have decreased strongly among older adults.

Our results regarding the socioeconomic differences in limitation trends are also in line with previous studies. Whereas most previous studies in the US found growing social inequalities in limitations (e.g. Schoeni et al., 2005; Tsai, 2016), studies in Europe pointed to mostly persisting socioeconomic differences over time (e.g. Moe & Hagen, 2011; Rubio-Valverde et al., 2021). Likewise, we found similarities in the age-dependent direction of trends across socio-economic groups. However, some differences emerged regarding the relative size of the observed trends. A more pronounced increase in limitations was observed for the younger age-group with more years of education. Contrarily, a more pronounced decrease in limitations was observed for older adults with fewer years of education. It must be noted, however, that a robustness analysis with an alternative operationalization of educational attainment did not replicate the socioeconomic difference in younger adults, as judged by the overlapping confidence intervals, whereas the robustness analysis replicated the difference in the relative strength of trends among older adults. Thus, our results point to persisting or, in the case of older adults, declining socio-economic differences over time in Europe, with mostly increasing limitations in younger age groups, mixed trends in the middle-aged groups and mostly decreasing limitations in the older age groups. It must be noted, however, that a direct comparison to previous studies is complicated by our empirical definition of the socio-economic groups. Due to the large cross-national ESS sample, only non-optimal, relatively broad distinctions between socioeconomic groups, such as ‘reported no income difficulties’ versus ‘reported income difficulties’, could be made. Clearly, more research on socioeconomic differences in age-differential trends in limitations is needed, especially studies accounting for education, income and occupational class differences simultaneously (Geyer, 2006).

### Potential Explanations for the Observed Trends

While numerous studies have explored why limitations have been decreasing in older adults, less studies have examined the reasons for the observed increases in limitations in middle-aged and younger adults. Regarding the decreases in limitations among older adults, studies point to the major importance of rising levels of education, decreasing levels of smoking, improving material conditions and medical advances (e.g. Chen & Sloan, 2015; Jagger et al., 2020; Jehn & Zajacova, 2018; Martin et al., 2010).

Regarding the increasing limitation trends among middle-aged and younger adults, there are several possible explanations for this increase, including medical advances and changing risk factors (Robine & Michel, 2004). First, in accordance to the failure of success hypothesis (Gruenberg, 1977), limitations in the middle-aged and young might be on the rise because medical advances have conquered humanities’ most debilitating diseases. Medical advances might have prolonged the life of those who, in earlier times, might have died due to illness, such as is the case with congenital heart disease in infants and children, but are now merely limited in their activities. Similarly, conditions that would have resulted in severe limitation, now might only lead to less-severe limitations, potentially explaining the especially pronounced increases over time for overall limitations (Beller et al., 2020).

Second, younger and middle-aged individuals might be exposed to increasing health risks over time (Stephan et al., 2019). Younger cohorts especially in Western Europe might have partly adopted unhealthy lifestyles including diminished levels of physical activity and reduced fruit and vegetable intake, resulting in ever-increasing levels of obesity and limitations in some subgroups. As one detailed example, the observed increase in limitations in the young and middle-aged might be related to the changing world of work. Most young and middle-aged adults spend a large part of their lives at work, which in recent years has increasingly involved large-scale changes regarding the nature of work, the performance of work and the use of new technologies (Goos, 2013; Henkens & van Solinge, 2021; Smedley et al., 2013). As a result, there is growing concern about the impact of potential changes in the world of work on the health development and physical functioning of working people (Heller et al., 2022; Neira, 2010). Accordingly, one recent study has reported increased work stress among younger and middle-aged adults in Europe (Rigó et al., 2021). Perhaps an increase in perceived difficulties to perform work tasks is one of the major factors contributing to increased rates of activity limitation in younger and middle-aged adults (Cabrero-García et al., 2020). Unfortunately, current empirical evidence on population-based trends in physical and psychosocial working conditions is scarce, mixed and difficult to interpret (Burr, 2021). Thus, clearly more studies are needed that empirically examine the origin of the increasing limitation trends in young and middle-aged adults and why they appear especially strong in some, but not other countries (Robine & Michel, 2004; Zajacova & Montez, 2018).

### Country-Specific Trends

The decreases and increases in limitations were not randomly distributed across countries but geographically clustered: Most increases were observed among countries in Western and Northern Europe, whereas most and strongest decreases were found in Eastern and Southern Europe. Perhaps, the potential changes in risk-factors were especially prominent among participants from Western and Northern Europe. In Germany, for example, Sperlich et al. (2020) recently found that disability rates as well as comorbid conditions such as obesity were increasing among people with diabetes. Similarly, medical advances in recent years might have been especially effective among older adults from Southern and Eastern Europe. For example, in Spain control of chronic diseases of which the prevalence is higher in older people has improved considerably (Banegas et al., 2015; Mata-Cases et al., 2016). As an additional explanation, limitation prevalences were generally higher in Eastern and Southern Europe, suggesting that ceiling and floor effects might also be one additional cause of the observed effects.

Not all countries confirmed to this geographical distinction. For example, in contrast to effects in countries such as Germany, Belgium and France, overall limitations decreased in all age groups in Austria. However, this is supported by the Austrian national statistics on life years with functional limitations, where also declines in functional limitations are generally found. According to our knowledge, no causal explanation for this development has been found yet. One possibility might be that risk factors for health have changed differently in Austria as compared to the other studied Western European countries. In line with this possibility, Großschädl and Stronegger (2013) reported that the prevalence of obesity in Austria remained rather stable at younger ages in contrast to other countries. However, it must be noted that they also found increasing trends of obesity similar to other countries among women and men aged 55 and older, such that this explanation is not applicable to the older age groups. As another example, in contrast to decreasing limitations in other Mediterranean countries, no significant trends were observed in Italy. According to statistics of the European Union, Italy ranks highly among European countries in life expectancy, yet when considering the self-perceived health and limitations after 65 years Italy falls close to the EU average (eurostat, 2022). Additionally, the Italian population has been one of the fastest ageing among developed countries, and it is estimated that in 2050, the share of over 65s will amount to 35.9% of the total population (United Nations, 2017). Consequently, if no further decreases in limitations among older adults can be achieved severe problems are to be expected regarding Italy’s population health. Especially in some countries from Northern Europe, such as Denmark, Ireland and Norway, older adults did not significantly improve over time, in contrast to other countries. One potential explanation for this might be a ceiling effect given that older adults in Northern Europe constitute among the healthiest populations of older adults globally (Mladovsky, 2009). Of course, these effects could also result from simple statistical uncertainty or data and measurement issues (Berger et al., 2015; Robine et al., 2013; Rubio-Valverde et al., 2019). However, it must be noted that at least the question wording has been constant across time points. Future studies are needed that study the mechanisms behind the differential trends across countries in more detail.

### Public Health Implications

From a practical perspective these results seem alarming. Although it certainly can be judged as a positive development that limitation rates appear to be declining in older adults, we also observed increasing limitations among middle-aged and young adults in several countries. If these increases in limitations prove to be chronic, it is also likely that they are accompanied by further disabilities and health ailments as these young and middle-aged populations age. Physical health is seen as more malleable during young and middle-age than in old age (Halfon et al., 2018). Therefore, special prevention efforts and assistance might be directed at younger and middle-aged cohorts to improve and maintain their health. These approaches might encompass individual, social, cultural and policy aspects (Tate & Pledger, 2003), such as assistive technology on an individual level, and less social isolation on the environmental level (Macdonald et al., 2018).

First, however, it should be examined how the overall life situation, health and well-being of people with limitations has changed over time. If, for example, increases in limitations were accompanied by improvements in the general health status, social participation and well-being of people with limitations, the trends among younger and middle-aged groups might not seem as daunting, as if increases in limitations were accompanied by a worsening health status, social participation and well-being. Examining the overall health status of people with limitations might also be important from a theoretical perspective. In addition to the compression and expansion of morbidity hypothesis, it had also been suggested that future increases in chronic conditions might be balanced by overall increases in quality of life of those with chronic conditions, suggesting a dynamic equilibrium of morbidity. Thus, future studies should analyse how the biopsychosocial health of people with limitations has changed over time (Molton & Jensen, 2010). Doing so would not only allow a more fine-grained judgement on trends in limitations but would also enable better targeted prevention and support efforts in practice.

### Limitations

The current study includes several limitations. The sample did not include institutionalized older adults and thus likely underestimates the true level of limitations in the population, especially among older adults. Additionally, aged care provision and its utilization differs between countries and over time, such that future studies are needed that examine trends in limitations in the institutionalized population (Khadka et al., 2019; Nagode & Lebar, 2019).

Furthermore, we only analysed a self-report measure as our dependent variable. The use of this indicator may not be a fully valid measure to compare the frequency of disability between countries. This could explain the heterogeneity of results from one country to another. For future studies, it might be beneficial to analyse other indicators and data sources of limitations and health, for example, by using insurance data (e.g. Safieddine et al., 2020; Tetzlaff et al., 2020).

Additionally, while half of the countries have consistently participated in every wave of the survey, there are also many gaps in the data. For example, Hungary only participated in three survey waves, and Greece only participated up to 2010. Therefore, the overall super-national prevalence estimates for each wave are highly dependent on the countries participating in that wave and interpretation about trends should mostly be considered on a national level such as presented in Figures 1 and 2*.* In a similar vein, response rates varied strongly between countries. While weighting was employed to mitigate this issue, it must be expected that certain sample selectivities, such as a good functional health bias, could not be completely controlled for (Beller et al., 2022). Given the declining response rates generally observed in population-based studies over time, this would suggest an under-estimation of increases in limitation over time, as participants with limitations are less likely to participate in research over time (Beller et al., 2022; Koen Beullens, 2018). As such, future studies could further investigate limitation trends with more complete datasets.

As another potential avenue for future research, socioeconomic differences in the strength of trends were observed regarding education. For example, declining trends of limitations in older adults were much more pronounced among those with fewer years of education and those with lower educational attainment. Future studies could further investigate the potential mechanisms for these differential trends.

Despite these limitations, we were able to comprehensively analyse how limitation rates of young, middle-aged and older age groups have changed over time in a large multinational sample. Future studies should replicate and expand upon these results.

## Appendix

**Table A1.** Overall Limitations and Severe Limitations Across Countries (European Social Survey Data From 2002–2018).

Country	Overall limitations (%)	Severe limitations (%)
Austria	20.49	3.77
Belgium	22.78	5.47
Bulgaria	17.32	4.00
Switzerland	18.69	3.14
Cyprus	17.02	4.97
Czechia	27.13	4.04
Germany	29.13	6.17
Denmark	25.55	5.08
Estonia	28.82	9.31
Spain	15.47	4.49
Finland	32.07	7.81
France	23.37	6.40
Great Britain	24.77	8.50
Greece	15.72	3.86
Croatia	22.46	5.57
Hungary	28.08	7.24
Ireland	16.09	3.46
Israel	19.36	5.46
Island	22.79	5.49
Italy	13.80	2.39
Lithuania	33.76	7.06
Netherlands	26.83	5.96
Norway	24.86	5.44
Poland	27.08	6.61
Portugal	19.25	4.12
Russia	31.31	5.58
Sweden	28.08	6.12
Slovenia	31.52	9.83
Slovakia	24.26	4.37
Ukraine	41.20	10.16

**Table A2.** Overall Limitations Across Time Periods and Within Countries (European Social Survey Data From 2002–2018).

Country	2002	2004	2006	2008	2010	2012	2014	2016	2018
Austria	22.89	22.50	23.57				20.93	20.32	23.98
Belgium	19.69	19.63	21.24	22.14	22.68	24.64	24.94	26.32	23.59
Bulgaria			21.75	19.43	17.60	20.20			17.70
Switzerland	20.05	19.77	21.74	19.99	18.08	18.58	21.06	17.91	19.83
Cyprus			9.78	16.74	22.00	20.31			23.68
Czechia	38.52	32.91		31.14	29.57	24.61	26.73	29.20	30.81
Germany	26.60	26.30	28.25	27.89	29.69	33.19	30.66	33.18	33.06
Denmark	24.09	21.71	25.50	26.78	25.43	24.57	27.35		28.71
Estonia		27.51	26.71	25.35	27.94	28.60	28.08	33.76	31.48
Spain	19.78	14.55	16.08	15.56	14.86	16.01	16.25	14.54	15.03
Finland	28.67	31.22	31.03	31.87	34.05	32.75	32.33	32.72	34.27
France			22.96	24.33	23.44	27.63	24.87	29.18	28.44
Great Britain	25.80	26.84	27.83	25.07	27.44	29.29	28.80	27.77	28.86
Greece	23.37	19.00		13.35	13.97				
Croatia				24.77	30.88				23.79
Hungary	30.20	30.18	33.87	30.36	30.56	25.74	29.68	27.02	22.49
Ireland	14.44	17.45	16.83	18.06	16.17	18.07	19.10	20.58	21.61
Israel	18.67			22.86	21.92	16.75	23.04	17.62	
Island		17.57				20.08		27.63	24.04
Italy	11.55					16.78		13.36	14.35
Lithuania					49.77	32.08	35.39	35.47	46.20
Netherlands	26.62	28.58	27.90	27.86	29.52	29.99	32.41	29.68	28.91
Norway	23.11	26.10	23.21	24.19	24.37	24.07	25.63	26.68	27.49
Poland	26.18	26.45	27.37	27.77	26.73	27.73	29.41	26.03	24.95
Portugal	15.79	18.94	23.43	21.68	19.42	20.31	21.38	22.91	22.21
Russia			40.57	38.45	34.05	30.92		30.60	
Sweden	27.19	27.73	27.95	27.15	27.36	26.62	30.42	30.63	28.08
Slovenia	32.51	34.30	32.82	30.24	30.19	25.97	35.26	31.11	30.81
Slovakia		22.24	22.69	25.23	30.09	26.07			36.38
Ukraine		47.41	45.24	45.02	42.52	39.55			

*Note.* Values represent percentages.

**Table A3.** Severe Limitations Across Time Periods and Within Countries (European Social Survey Data From 2002–2018).

Country	2002	2004	2006	2008	2010	2012	2014	2016	2018
Austria	3.91	4.70	3.80				5.12	4.24	4.79
Belgium	4.22	4.01	5.39	5.61	5.35	5.53	5.68	6.54	6.91
Bulgaria			5.21	4.43	4.23	4.89			3.77
Switzerland	3.39	3.55	3.25	3.36	2.98	3.31	3.84	2.33	3.70
Cyprus			2.87	3.87	7.23	6.80			6.08
Czechia	8.22	7.21		5.90	5.62	4.07	3.91	3.64	5.01
Germany	5.52	6.30	5.95	5.60	6.39	7.12	6.86	6.76	7.30
Denmark	4.38	4.62	4.91	5.42	5.91	5.05	5.24		5.06
Estonia		10.54	7.12	9.13	8.85	11.36	9.86	8.18	8.41
Spain	5.73	4.14	4.79	4.49	4.46	4.88	5.11	3.92	3.77
Finland	7.86	7.54	7.60	8.06	9.00	7.32	6.82	8.60	7.71
France			6.09	7.00	6.41	7.92	7.02	8.70	8.85
Great Britain	8.72	9.77	11.11	9.64	9.29	9.97	10.15	10.66	10.38
Greece	6.05	4.39		2.44	4.18				
Croatia				7.49	7.87				6.16
Hungary	9.53	8.93	10.39	8.91	9.09	5.20	6.20	5.25	4.64
Ireland	3.42	3.67	3.57	3.96	3.22	3.48	3.83	5.18	5.50
Israel	4.58			5.60	5.81	5.44	7.03	5.77	
Island		4.53				4.74		6.44	5.98
Italy	2.67					1.98		2.64	2.18
Lithuania					16.94	6.05	7.23	7.44	10.10
Netherlands	5.81	6.81	6.63	6.24	6.96	7.73	7.88	6.26	6.51
Norway	4.53	6.10	4.47	5.45	5.78	5.51	6.22	5.73	5.60
Poland	6.88	7.28	6.92	8.01	5.98	5.91	6.75	5.29	5.65
Portugal	2.64	3.59	6.57	5.08	4.26	4.06	4.58	4.39	5.09
Russia			9.14	9.38	6.50	5.67		4.99	
Sweden	6.82	6.28	7.55	5.41	5.50	5.14	5.61	7.18	5.36
Slovenia	10.37	9.40	11.38	10.31	9.65	7.85	11.64	9.54	8.11
Slovakia		4.48	4.59	4.37	7.27	4.00			4.86
Ukraine		13.96	12.14	12.96	12.9	9.50			

*Note.* Values represent percentages.

**Table A4.** Overall Limitations Across Time Periods and Within Countries in the Young Age Group (European Social Survey Data From 2002–2018).

Country	2002	2004	2006	2008	2010	2012	2014	2016	2018
Austria	10.96	11.53	10.91				9.48	6.95	7.75
Belgium	10.40	9.28	14.26	9.75	10.68	12.52	13.55	16.17	14.35
Bulgaria			6.00	4.22	4.18	2.74			3.12
Switzerland	9.80	9.69	9.64	10.99	7.52	10.19	12.75	10.80	11.81
Cyprus			2.56	5.28	8.36	5.99			8.02
Czechia	10.54	12.02		11.67	9.29	9.82	9.31	11.33	10.60
Germany	11.14	12.10	13.83	13.56	14.90	17.95	16.67	22.51	18.45
Denmark	15.25	15.02	17.00	16.50	20.80	17.37	22.46		18.05
Estonia		7.77	9.98	10.93	10.33	19.02	11.33	14.20	15.17
Spain	4.17	5.17	4.62	3.93	2.23	6.72	6.36	6.03	5.76
Finland	15.90	16.26	16.39	17.15	19.69	18.09	18.96	21.49	25.97
France			11.91	13.35	11.17	12.88	14.79	15.00	14.08
Great Britain	12.12	11.62	14.07	12.31	12.38	12.87	14.65	15.05	15.73
Greece	5.81	3.97		4.83	3.64				
Croatia				8.68	7.42				5.39
Hungary	9.64	9.68	12.45	9.41	10.68	8.92	7.79	7.40	3.46
Ireland	7.82	6.34	10.32	10.10	7.96	8.76	9.20	8.27	11.78
Israel	7.90			7.69	6.77	5.19	8.15	6.75	
Island		11.69				15.48		21.89	21.80
Italy	3.80					6.90		3.18	2.55
Lithuania					20.15	5.87	8.91	7.72	11.61
Netherlands	16.05	17.40	15.06	17.49	18.77	17.20	18.46	17.58	16.70
Norway	14.93	16.22	14.89	15.31	13.11	15.81	18.22	17.38	20.45
Poland	8.45	9.80	8.64	9.74	9.37	11.43	10.95	9.19	9.50
Portugal	4.81	4.60	8.07	5.44	5.05	6.62	10.31	8.57	9.09
Russia			16.46	15.09	10.43	13.28		10.34	
Sweden	17.06	15.37	16.10	15.12	19.65	15.83	20.93	19.78	21.70
Slovenia	14.33	13.78	10.47	10.59	11.79	9.35	14.11	12.29	12.38
Slovakia		10.89	9.79	7.29	10.11	7.72			13.54
Ukraine		19.02	19.27	20.33	16.30	16.71			

*Note.* Values represent percentages.

**Table A5.** Severe Limitations Across Time Periods and Within Countries in the Young Age Group (European Social Survey Data From 2002–2018).

Country	2002	2004	2006	2008	2010	2012	2014	2016	2018
Austria	1.51	2.51	1.72				2.03	1.78	0.88
Belgium	1.50	1.41	1.63	2.55	1.88	1.91	1.83	2.25	2.93
Bulgaria			0.50	0.65	1.45	0.39			0.45
Switzerland	1.36	1.38	1.07	1.59	0.61	0.93	1.44	0.95	1.29
Cyprus			1.42	0.78	2.51	2.49			0.53
Czechia	1.20	2.08		1.36	1.43	1.47	0.97	0.49	1.26
Germany	1.96	1.44	2.96	1.62	2.82	3.37	2.54	3.46	3.16
Denmark	2.30	2.85	2.01	3.05	3.60	1.93	3.45		3.25
Estonia		0.67	1.66	2.61	1.75	7.98	1.94	2.49	1.79
Spain	0.83	0.70	1.19	1.47	0.13	1.90	0.45	1.60	0.36
Finland	2.37	1.36	3.03	3.01	3.31	2.10	1.62	3.88	3.90
France			2.15	3.00	1.75	2.50	3.22	3.28	3.35
Great Britain	2.75	2.94	5.06	4.06	3.95	3.57	2.90	4.50	4.49
Greece	1.32	0.72		1.21	1.28				
Croatia				1.89	1.69				0.80
Hungary	1.56	2.33	2.15	1.05	2.84	0.68	0.76	0.21	0.65
Ireland	1.86	0.79	2.06	2.05	1.19	1.32	1.66	1.51	1.58
Israel	2.52			2.76	1.83	1.04	2.26	2.18	
Island		3.46				0.97		4.71	5.26
Italy	0.48					0.94		0.61	0.27
Lithuania					4.13	0.29	1.06	0.96	1.42
Netherlands	3.09	3.55	2.64	2.12	2.37	3.60	2.56	2.42	2.25
Norway	1.84	2.84	1.61	1.95	2.52	3.06	3.04	3.44	3.48
Poland	0.86	1.63	1.08	2.47	0.67	1.17	0.82	1.13	1.49
Portugal	0.92	0.97	1.64	1.47	0.97	1.16	0.56	1.43	1.45
Russia			1.25	1.13	0.81	1.51		0.87	
Sweden	2.97	2.31	3.55	1.45	3.14	1.98	2.49	3.37	4.95
Slovenia	2.00	1.59	1.50	2.04	2.85	1.17	2.48	1.45	0.95
Slovakia		0.61	1.83	1.50	0.95	1.23			2.62
Ukraine		2.62	3.21	3.67	2.06	2.41			

*Note.* Values represent percentages.

**Table A6.** Overall Limitations Across Time Periods and Within Countries in the Middle-Aged Group (European Social Survey Data From 2002–2018).

Country	2002	2004	2006	2008	2010	2012	2014	2016	2018
Austria	22.93	23.30	24.71				18.91	18.74	21.27
Belgium	20.83	21.04	20.21	25.23	26.19	25.34	27.01	28.02	26.40
Bulgaria			20.30	17.55	13.82	15.32			10.30
Switzerland	20.02	21.02	20.59	19.36	19.00	18.14	21.78	17.82	19.27
Cyprus			9.07	16.56	17.27	20.40			17.68
Czechia	34.64	34.44		34.85	31.78	23.21	25.68	31.54	31.45
Germany	27.80	26.75	26.10	26.93	31.56	35.40	31.56	33.89	34.40
Denmark	26.69	23.48	25.14	28.50	24.72	24.17	28.73		31.00
Estonia		26.19	25.65	23.54	26.19	26.81	25.64	34.10	28.33
Spain	15.96	13.76	15.38	11.96	14.90	16.92	14.37	12.26	13.03
Finland	28.32	32.90	31.20	33.26	36.04	35.24	32.57	31.65	34.13
France			25.06	23.94	23.37	26.35	25.90	29.10	29.28
Great Britain	26.32	29.78	27.03	25.23	26.91	28.39	27.58	25.97	28.32
Greece	19.96	13.23		12.23	9.25				
Croatia				23.47	26.65				23.03
Hungary	35.09	35.63	34.16	32.78	33.75	26.93	27.36	20.27	18.17
Ireland	15.59	17.55	17.46	20.41	17.81	19.09	18.63	20.07	22.71
Israel	20.77			24.81	23.54	18.37	23.06	16.97	
Island		20.83				21.89		28.57	23.61
Italy	11.52					15.73		10.64	10.24
Lithuania					48.57	31.92	32.73	30.96	42.11
Netherlands	28.89	29.47	29.10	27.93	30.64	32.31	34.43	31.42	30.79
Norway	25.50	29.06	26.24	25.52	30.09	27.30	24.31	29.26	29.68
Poland	32.97	33.73	35.76	31.35	30.45	29.54	32.56	27.77	20.77
Portugal	13.83	17.70	21.50	17.17	16.67	16.24	20.38	22.85	19.73
Russia			42.55	38.06	38.48	32.11		32.09	
Sweden	29.57	32.56	29.90	28.12	26.75	28.99	29.06	29.79	23.17
Slovenia	38.41	36.95	38.37	29.72	31.99	25.09	37.40	33.56	28.44
Slovakia		24.19	23.98	23.65	28.55	26.07			29.75
Ukraine		49.29	48.42	48.63	43.63	41.59			

*Note.* Values represent percentages.

**Table A7.** Severe Limitations Across Time Periods and Within Countries in the Middle-Aged Group (European Social Survey Data From 2002–2018).

Country	2002	2004	2006	2008	2010	2012	2014	2016	2018
Austria	4.23	3.92	3.44				4.02	3.55	5.22
Belgium	4.69	4.49	4.89	6.37	5.46	6.24	6.68	7.42	8.24
Bulgaria			4.36	3.59	2.96	3.41			3.02
Switzerland	3.58	4.09	3.19	4.51	3.53	3.94	4.93	2.37	4.30
Cyprus			3.10	2.90	6.33	5.76			2.89
Czechia	6.25	7.01		5.79	5.45	3.40	2.94	3.97	6.13
Germany	6.46	5.88	5.08	5.53	6.56	8.44	6.41	7.74	7.32
Denmark	4.60	5.35	5.79	5.73	5.87	5.66	5.08		6.07
Estonia		10.06	6.46	8.15	8.40	10.99	8.55	7.71	7.05
Spain	4.42	4.18	3.69	3.07	4.30	4.48	4.42	2.90	3.82
Finland	8.28	7.99	6.89	7.05	9.52	7.78	5.94	8.23	6.74
France			5.98	6.56	6.92	7.02	7.56	9.85	8.31
Great Britain	8.94	9.13	11.23	9.26	8.44	8.78	10.32	9.17	10.07
Greece	4.04	3.25		2.58	3.08				
Croatia				5.08	4.90				4.96
Hungary	10.26	10.09	9.45	8.70	9.29	5.15	5.36	1.98	3.90
Ireland	3.12	4.36	4.29	5.14	3.82	3.45	4.43	5.39	7.39
Israel	5.32			6.31	6.48	5.93	6.35	6.00	
Island		2.92				7.41		6.88	5.56
Italy	2.60					1.07		1.68	1.48
Lithuania					14.65	4.83	5.47	4.26	6.27
Netherlands	6.34	7.42	7.01	6.36	8.19	8.84	9.20	6.31	8.32
Norway	5.90	6.60	4.82	6.93	7.60	6.96	6.69	6.26	5.90
Poland	8.57	7.76	7.86	7.37	5.71	4.37	6.36	4.46	3.68
Portugal	1.77	2.39	4.75	3.72	4.14	3.86	3.61	4.31	3.14
Russia			6.15	6.29	5.38	4.37		3.83	
Sweden	7.36	8.11	7.48	5.54	5.55	5.41	5.46	6.97	4.26
Slovenia	12.42	8.00	12.85	10.24	9.88	6.41	11.07	10.1	6.88
Slovakia		5.05	2.88	3.27	6.18	4.06			4.03
Ukraine		11.77	9.90	11.26	11.52	7.45			

*Note.* Values represent percentages.

**Table A8.** Overall Limitations Across Time Periods and Within Countries in the Old Age Group (European Social Survey Data From 2002–2018).

Country	2002	2004	2006	2008	2010	2012	2014	2016	2018
Austria	50.74	51.83	51.87				41.06	41.34	44.98
Belgium	39.86	39.32	36.86	40.24	39.23	44.65	41.69	41.60	33.85
Bulgaria			43.60	38.38	34.28	39.75			36.18
Switzerland	40.16	35.54	40.59	35.15	33.01	34.31	33.95	29.12	34.59
Cyprus			26.47	47.18	50.63	44.49			42.25
Czechia	72.62	64.35		64.65	64.10	51.72	61.28	61.70	57.01
Germany	52.35	52.14	54.94	48.93	49.04	49.77	46.47	48.29	50.09
Denmark	37.55	30.95	39.38	37.68	33.72	35.08	32.34		37.99
Estonia		62.25	53.46	53.35	55.76	44.56	53.12	59.72	55.15
Spain	46.89	38.16	42.49	44.42	44.07	31.92	37.14	31.51	35.06
Finland	57.10	54.48	53.41	53.63	51.50	48.03	46.34	48.39	43.65
France			38.08	43.41	42.29	45.28	36.56	43.17	41.31
Great Britain	46.24	46.91	48.44	43.69	47.64	46.66	43.97	43.58	42.56
Greece	49.66	44.05		38.15	39.67				
Croatia				55.67	64.41				44.85
Hungary	61.97	59.77	57.63	61.22	62.75	55.30	60.56	58.66	48.65
Ireland	27.42	36.55	28.80	28.16	28.96	33.27	34.06	37.20	29.06
Israel	50.90			56.27	51.74	41.8	48.02	37.24	
Island		24.69				28.18		35.20	27.62
Italy	27.86					38.41		32.93	32.21
Lithuania					77.63	68.79	70.80	74.77	71.52
Netherlands	40.50	43.32	46.75	43.16	40.14	39.29	44.63	39.87	42.67
Norway	37.58	43.56	35.42	42.40	34.62	34.17	41.78	38.49	35.27
Poland	60.95	63.84	58.78	64.84	64.49	61.01	58.92	54.10	58.18
Portugal	34.77	38.37	42.14	40.43	32.56	38.86	32.13	36.59	37.87
Russia			78.49	76.77	72.99	65.97		66.88	
Sweden	44.79	41.38	47.77	46.02	39.02	39.03	44.12	42.42	39.28
Slovenia	65.34	70.57	63.12	66.79	59.85	54.96	61.48	53.10	58.82
Slovakia		60.00	61.64	51.71	59.11	54.64			59.66
Ukraine		77.36	75.83	75.50	72.37	72.79			

*Notes.* Values represent percentages.

**Table A9.** Severe Limitations Across Time Periods and Within Countries in the Old Age Group (European Social Survey Data From 2002–2018).

Country	2002	2004	2006	2008	2010	2012	2014	2016	2018
Austria	8.55	13.62	10.09				11.59	8.87	8.10
Belgium	9.80	8.67	13.71	10.06	12.22	10.44	10.70	12.67	11.11
Bulgaria			12.50	9.76	8.40	10.25			6.78
Switzerland	6.91	6.38	6.36	3.96	5.56	6.21	5.86	4.41	6.60
Cyprus			5.29	14.36	15.90	16.10			13.95
Czechia	18.45	16.18		16.31	14.22	9.81	11.54	9.57	7.95
Germany	9.93	16.54	12.40	11.04	11.66	9.62	13.14	9.92	12.74
Denmark	8.57	6.35	7.19	8.12	9.38	8.12	8.31		5.64
Estonia		27.59	16.34	22.07	19.64	16.58	21.93	16.67	18.13
Spain	14.35	12.17	14.74	12.85	14.89	11.58	14.55	9.43	9.45
Finland	18.75	17.68	16.00	18.68	16.40	13.51	13.76	15.06	13.32
France			13.47	14.55	12.50	15.09	11.18	12.31	14.97
Great Britain	17.63	21.97	19.38	18.64	17.57	17.93	16.79	19.17	16.61
Greece	14.48	10.19		5.23	11.00				
Croatia				22.00	19.61				13.95
Hungary	24.59	19.92	21.07	22.45	20.92	13.95	14.32	15.70	9.87
Ireland	8.06	7.13	5.18	5.26	5.98	7.56	5.83	9.52	6.33
Israel	9.94			11.06	13.18	15.24	16.01	11.56	
Island		12.35				8.18		8.38	7.62
Italy	7.46					6.10		7.32	5.12
Lithuania					31.40	16.10	17.35	20.04	20.13
Netherlands	9.50	10.33	12.47	12.11	9.98	10.08	11.57	10.23	9.07
Norway	7.64	13.26	10.70	10.00	8.39	7.19	10.96	8.83	8.73
Poland	20.32	25.89	20.99	23.44	21.01	20.24	19.11	14.89	16.04
Portugal	6.47	8.33	14.15	9.63	6.70	7.11	9.18	7.32	11.30
Russia			27.52	27.95	20.41	17.59		15.09	
Sweden	14.37	10.08	15.92	11.94	8.67	9.47	9.69	11.21	7.01
Slovenia	27.09	27.66	26.95	25.46	21.56	21.76	25.80	20.00	19.50
Slovakia		17.65	18.53	10.24	17.73	8.20			7.39
Ukraine		30.19	27.71	30.00	28.04	24.62			

*Note.* Values represent percentages.

**Table A10.** Multilevel Logistic Regression Results Predicting Overall Limitations Across Time According to Educational Attainment (European Social Survey Data From 2002–2018).

Lower education					Higher education				
Young age (ages 15–39)									
*N* = 74909	OR	CI	Z	*p*	*N* = 40983	OR	CI	z	*p*
Age	1.45	[1.41, 1.49]	24.72	< .001	Age	1.21	[1.14, 1.28]	6.13	< .001
Gender (Female)	1.23	[1.18, 1.29]	9.31	< .001	Gender (Female)	1.16	[1.09, 1.24]	4.50	< .001
Trend	1.52	[1.38, 1.67]	8.51	< .001	Trend	1.36	[1.17, 1.57]	4.03	< .001

Middle age (ages 40–64)									
*N* = 89361	OR	CI	Z	*p*	*N* = 52635	OR	CI	z	*p*
Age	1.63	[1.6, 1.67]	44.98	< .001	Age	1.61	[1.56, 1.66]	30.89	< .001
Gender (Female)	1.14	[1.11, 1.17]	8.56	< .001	Gender (Female)	1.26	[1.21, 1.32]	10.38	< .001
Trend	0.99	[0.93, 1.06]	−0.30	.761	Trend	1.09	[0.98, 1.20]	1.65	.100

Older age (ages 65+)									
*N* = 58685	OR	CI	Z	*p*	*N* = 18229	OR	CI	z	*p*
Age	1.81	[1.76, 1.86]	41.43	< .001	Age	1.77	[1.68, 1.87]	20.98	< .001
Gender (Female)	1.2	[1.16, 1.25]	9.82	< .001	Gender (Female)	1.12	[1.04, 1.19]	3.21	< .001
Trend	.76	[0.70, 0.82]	−6.69	.001	Trend	0.86	[.74, 1.00]	−1.90	.058

*Note.* A reduced sample size of *N* = 334,802 needed to be used for this analysis because of missing values in the educational attainment variable.
